# The qualitative dimension of Nursing workload: a measurement proposal*

**DOI:** 10.1590/1518-8345.3274.3238

**Published:** 2019-12-05

**Authors:** Danielle Fabiana Cucolo, Marcia Galan Perroca

**Affiliations:** 1 Universidade Federal de São Carlos, São Carlos, SP, Brazil.; 2 Faculdade de Medicina de São José do Rio Preto, Departamento de Enfermagem Especializada, São José do Rio Preto, SP, Brazil.

**Keywords:** Workload, Hospital Nursing Staff, Nursing Assessment, Hospital Administration, Health Management, Assessment on Outcome and Process (Health Care), Carga de Trabalho, Equipe de Enfermagem do Hospital, Avaliação em Enfermagem, Administração Hospitalar, Gestão em Saúde, Avaliação de Processos e Resultados (Cuidados de Saúde), Carga de Trabajo, Personal de Enfermería del Hospital, Evaluación en Enfermería, Administración Hospitalaria, Gestión em Salud, Evaluación de Resultados y Procesos (Atención de Salud)

## Abstract

**Objective:**

construct and test a proposal to measure the qualitative dimension of nursing workload; identify the workload cut-off point and its indicator as predictors of the good and optimal nursing care product score.

**Method:**

this is a descriptive study conducted in four inpatient units and four intensive care units of a Brazilian teaching hospital, considering 308 evaluations performed by 19 nurses. Four measurement instruments were used: three to assess the care demand in relation to nursing and the other to classify the care product delivered at the end of the shift. The workload was calculated and its indicator was constructed.

**Results:**

a weak and inverse correlation was found between the care product score, workload and the workload indicator and the workload indicator in the units and moderate and inverse between Nursing care planning and Care needs assistance with the number of hospitalized patients.

**Conclusion:**

it is possible to associate workload and its indicator with the care product. Nursing workload ≤ 173 hours (24 hours) and indicator ≤ 12.3 hours / professional were associated with a higher probability of obtaining a “good” and “optimal” score in the care product in the inpatient units.

## Abstract (pt)

**Objetivo:**

construir e testar uma proposta para mensurar a dimensão qualitativa da carga de trabalho em Enfermagem; identificar o ponto de corte da carga de trabalho e seu indicador como preditores do escore produto do cuidar em Enfermagem bom e ótimo.

**Método:**

estudo descritivo realizado em quatro unidades de internação e quatro unidades de terapia intensiva de um hospital de ensino brasileiro, considerando-se 308 avaliações realizadas por 19 enfermeiros. Foram utilizados quatro instrumentos de mensuração: três para avaliar a demanda de cuidados em relação à Enfermagem e o outro para classificar o produto do cuidar entregue ao final do plantão. Calculou-se a carga de trabalho e foi construído o seu indicador.

**Resultados:**

encontrou-se correlação fraca e inversa entre o escore produto do cuidar, carga de trabalho e o indicador carga de trabalho nas unidades; moderada e inversa entre Planejamento da assistência de Enfermagem e Atendimento das necessidades assistenciais com o número de pacientes internados.

**Conclusão:**

é possível associar carga de trabalho e seu indicador ao produto do cuidar. Carga de trabalho em Enfermagem ≤ 173horas (24 horas) e indicador ≤ 12,3horas/profissional associaram-se com maior probabilidade de obtenção de escore “bom” e “ótimo” no produto do cuidar nas unidades de internação.

## Abstract (es)

**Objetivo:**

construir y comprobar una propuesta para medir la dimensión cualitativa de la carga de trabajo en Enfermería; identificar el punto de corte de la carga de trabajo y su indicador como predictores del puntaje proveniente del cuidar en Enfermería: bueno y óptimo.

**Método:**

estudio descriptivo realizado en cuatro unidades de internación y cuatro unidades de terapia intensiva de un hospital de enseñanza brasileño; fueron examinadas 308 evaluaciones realizadas por 19 enfermeros. Se utilizaron cuatro instrumentos de medición: tres para evaluar la demanda de cuidados en relación a la Enfermería y otro para clasificar el producto del cuidar entregado al final del turno. Se calculó la carga de trabajo y fue construido su indicador.

**Resultados:**

se encontró correlación débil e inversa entre el puntaje producto del cuidar, carga de trabajo y el indicador carga de trabajo en las unidades; esta fue moderada e inversa entre Planificación de la asistencia de Enfermería y Atención a necesidades asistenciales con el número de pacientes internados.

**Conclusión:**

es posible asociar la carga de trabajo y su indicador al producto del cuidar. La carga de trabajo en Enfermería ≤ 173horas (24 horas) e el indicador ≤ 12,3horas/profesional se asociaron con mayor probabilidad de obtención de puntaje “bueno” y “óptimo” en el producto del cuidar, en las unidades de internación.

## Introduction

Nursing workload (NWL) can be conceived as the amount of time, physical and cognitive effort required of professionals to perform direct, indirect and non-patient care activities^(^ 1 ^)^. This approach broadens the concept of time devoted to patient demands, including the various actions taken by staff regarding the practice environment and professional development^(^ 2 ^)^.

This is a complex phenomenon that must be evaluated by nurses considering, in addition to the care needs required by patients, determining factors concerning the organization, the unit, the team, the individual (professional, patient and family) and the care system^(^ 3 ^)^. Among the factors that have the greatest impact on NWL, Finnish nurses emphasized work organization: insufficient staff and task planning; working conditions: inadequate resources and telephone requests and the skills needed to manage demands^(^ 4 ^)^; already Belgian researchers^(^ 5 ^)^ identified interruptions during activities, patient turnover and mandatory records. It is important to highlight that some factors do not directly affect NWL, but compromise the dynamics of their work and are perceived subjectively by nurses^(^ 3 ^)^.

Thus, the following attributes of NWL include: time spent in activities; the qualification of the team; the care needs of the patient; the physical, mental and emotional commitment of professionals, including work adaptability^(^ 2 ^)^. Researchers also advocate for the management of human factors and the engineering of systems and work processes that interact dynamically influencing NWL, the quality of services provided and the safety of patients and health professionals^(^ 6 ^-^ 7 ^)^. 

This management model would enable the identification of risks, such as missing care, based on early warning such as the high number of patients per nurse, among other inadequacies in the practice context^(^ 8 ^)^. Above all, the overload can compromise the attendance of the required activities during the shift, generating exhaustion and professional dissatisfaction and adverse events with the patients^(^ 9 ^)^. 

When investigating nurses’ daily workload using the RAFAELA system^(^ 10 ^)^, there was a greater chance of incidents (10% to 30%) and patient mortality (40%) when the values are above the ideal level and, conversely, this probability reduces 25%. It is inferred, therefore, that by assuming less workload, the nurse will have more time for care, preventing preventable clinical deterioration and patient incidents^(^ 10 ^)^.

Other studies corroborate the findings regarding the reduction of patient survival due to exposure to nursing work overload^(^ 11 ^-^ 12 ^)^, in addition to the risks associated with caring for different occupational categories / qualifications^(^ 11 ^)^. Hospitals that hold 60% of nurses on staff and scale up to six patients per nurse have 30% lower mortality than those in which the nurse cares, on average, eight patients and represents only 30% of the nursing staff^(^ 11 ^)^. 

Given these results, NWL measurement systems have been disseminated to determine the amount of personnel needed to meet the care demands^(^ 1 ^)^ and thus allocate resources appropriately^(^ 10 ^)^. In addition to instrumentalizing nurses in daily staff sizing and administrative negotiations, they can also support clinical evaluations and decisions involving process improvement^(^ 13 ^)^. 

However, the multifactorial etiology of NWL is not included in these instruments and the numerical value obtained from the classifications and the relationship with the time spent does not help the manager in the development of preventive actions and knowledge of the quality of care provided.

A new management method for NWL is being developed in the Netherlands to balance the needs of patients with the quantitative and qualitative framework of the nursing staff. This protocol aims to obtain the time required according to the patients’ characteristics, the activities performed and the average time dedicated, as well as the perception of emotional, physical and mental burdens^(^ 14 ^)^. But pretending to contemplate all the attributes of NWL in one instrument can be difficult^(^ 5 ^)^.

Recently, a scale for Nursing Care Product Evaluation (APROCENF, in Portuguese) was developed and validated based on the structural factors and methods of work organization^(^ 15 ^)^. This scale makes it possible to identify critical aspects in the nursing care system that may influence the product delivered at the end of the shift, classified as: poor, fair, good or optimal. It is important to emphasize that APROCENF does not evaluate the performance of nurses or staff, but the factors and methods that contribute, positively or negatively, to professional practice^(^ 15 ^)^. 

Thus, given the various demands required of the nurse, APROCENF could contribute to the identification of risks inherent to the practice environment (available resources, in-service education, teamwork), care planning and monitoring, patient / family care and mitigation of incidents perceived by nurses from the work dynamics. This inference meets the management of human factors and process analysis and was therefore considered in this study as the qualitative dimension of NWL. This research is linked to the research group Management of Health and Nursing Services (GESTSAÚDE) and proposes to answer the following questions: *Is it possible to associate NWL and the nursing workload indicator (NWLi) with the care product? Is there a correlation between numerical variables (number of nurses and nursing technicians / assistants, total nursing professionals, total patients, total beds, occupancy rate, NWL and NWLi) with APROCENF scores? What is the cut-off point of NWL and NWLi as predictors of the good and optimal nursing care product score?*

To this end, the following objectives were outlined: to construct and test a proposal to measure the qualitative dimension of the nursing workload and to identify the cut-off point of NWL and NWLi as predictors of the good and optimal nursing care product score. 

## Method

Descriptive, cross - sectional study of quantitative design conducted in four inpatient units (IUs) and four intensive care units (ICUs) of a hospital in Campinas - interior of the state of São Paulo Two of the IUs were Medical-Surgical clinics, one Surgical and one Pediatric, totaling 109 beds; ICUs - General, Coronary, Pediatric and Neonatal - corresponded to 71 beds. 

To define the sample size (evaluations of shifts), the method of comparing categories of the APROCENF score between the IUs and ICUs was used, setting the significance level at 5% and power at 80%. It was estimated that a sample of N = 294 evaluations of shifts (n = 147 for each unit) would be representative for the comparison between two groups^(^ 16 ^)^.

This investigation is part of the project “Workload and its influence on the results of the care process”, approved by the institution’s Research Ethics Committee (Process No. 0379/2011), and, depending on the results of the first objectives, was performed later. 

Nurses (n = 19) from different work shifts, working in the respective units during the data collection period, were invited, for convenience, and advised to apply APROCENF. The evaluations were conducted between January and September 2014. In early 2017, data were available in the institution’s computerized system of staffing and patient classification in the units, considering the dates of the evaluations. 

To reach the proposed objectives, four measurement instruments were applied: APROCENF scale^(^ 15 ^)^; Two Patient Classification Instruments (PCIs) (one for adult^(^ 13 ^)^ and another pediatric^(^ 17 ^)^) and the Nursing Activities Score (NAS)^(^ 18 ^)^. It is noteworthy that the first three instruments^(^ 13 ^,^ 15 ^,^ 17 ^)^ were constructed and had their psychometric properties tested in Brazil, respectively in 2017, 2013 and 2014, and the last^(^ 18 ^)^, was validated for Brazilian culture in 2009.

 APROCENF consists of eight items: 1. Nursing care planning; 2. Resources needed to provide care; 3. Nursing staff sizing; 4. Educational actions and professional development; 5. Monitoring and transfer of care; 6. Interaction and multidisciplinary action; 7. Attention to the patient and / or family member and 8. Meeting the care need.

Each item includes four graduations (1 to 4), representing, increasingly, the best product of nursing care. The nurse should analyze all items at the end of the shift, identifying the option that most closely matches the professional practice. After evaluating all the items, the graduated scores are added and the product delivered by Nursing will be classified as: Poor (eight to 12 points), Fair (13 to 20 points), Good (21 to 28 points) or Optimal (29 to 32 points)^(^ 15 ^)^.

The new version of PCI^(^ 13 ^)^, in its nine areas of care, incorporates the opinion of nurses / users and new nursing practices, in line with advances in health. Each area is composed of four degrees, being “1” the lowest demand for patient care in relation to nursing care and “4” the highest. The nurse must evaluate each area of care, choosing the condition that most closely matches that patient. The values are summed and the type of care, classified as: Minimum (MC) (9-12 points), Intermediate (IC) (13 to 18 points), Semi-intensive (SI) (19 to 24 points) or Intensive (In) (25 to 36 points). Similarly, pediatric PCI^(^ 17 ^)^ directs evaluation for family, patient and therapeutic procedures with 11 care areas, classifying them into MC (11 to 17 points), IC (18 to 23), High Dependency (HD) (24 to 30), SI (31 36), and In (37 to 44).

The NAS is indicated for use in ICUs, measuring the time devoted by nursing in patient care 24 hours. This instrument consists of seven categories: basic activities; ventilatory support; cardiological; renal; neurological; metabolic rate and specific interventions, and 23 items with a representative score of care needs^(^ 18 ^)^. The nurses then identify the items corresponding to each patient’s demand and the sum of the points reflects the time spent (in percentage) by the nursing team in direct and indirect care activities.

The research included four steps: 1. Evaluation of the care product in IUs and ICUs; 2. Measurement of NWL; 3. Construction and calculation of NWLi in these units and 4. Association between NWL and NWLi with the care product score. 

For the operationalization of the APROCENF scale, each participant performed between 11 and 20 evaluations, depending on the number of nurses per unit. The registration in printed form was done in the final moments of the shift. The determining factors for the product of nursing care represented, in this study, a portrait of the unit at 24 hours. Participants were also asked to answer a questionnaire for demographic and professional characterization.

Data sheets with the daily classification of patients in relation to dependence on nursing care in the IU and ICU were retrospectively verified through a computerized institutional system, considering the days when APROCENF was applied. This classification has been instituted for more than five years in the practice of nurses of this service and is performed daily at night, using a PCI for the Adult IU ^(^ 13 ^)^ and another for the Pediatric unit^(^ 17 ^)^ and the NAS in the ICUs. 

Access to the classification of patients from the ICUs allowed for the knowledge of the variables that make up the measurement of NWL. However, in addition to obtaining the number of patient-days per care category, it was necessary to associate the hours dedicated by Nursing in the 24 hours, and thus considered: MC - four hours; IC - six hours, SI - ten hours, HD - ten hours and In - 18 hours ^(^ 19 ^)^. For the calculation of daily NWLin the IUs, the following equation was adopted^(^ 19 ^)^:

$$\begin{array}{c} {\mathrm{NWL}}_{\mathrm{IUs}}=(nº\;\mathrm{MC}\;\times \;4)+(nº\;\mathrm{IC}\;\times \;6)+(nº\;\mathrm{SI}\;\times \;10)+\\ (nº\;\mathrm{HD}\;\times \;10)+(nº\;\mathrm{In}\;\times \;18)\\ \mathrm{in}\;\mathrm{which}\;nº=\mathrm{number}\;\mathrm{of}\;\mathrm{patient}\;\mathrm{days}\;\mathrm{per}\;\mathrm{care}\;\mathrm{category} \end{array}$$

Importantly, the data sheet used by nurses to measure NWL in ICUs was programmed to convert NAS points into hours, ie when entering the NAS point (percentage), the value was automatically divided by 100 and multiplied by 24. In this case, the values related to the hours required by each patient on a given day (corresponding to the application of APROCENF) were summed by the researcher and the daily NWL was reached for the ICUs:

$${\mathrm{NWL}}_{\mathrm{ICUs}}=\Sigma \mathrm{NAS}(\mathrm{hours})$$

The daily occupancy rate of the IUs and ICUs was also obtained considering the number of patient-days and the total active beds in each unit. 

In the third stage of this study, the researchers proposed a new indicator of nursing workload (NWLi), considering the NWL (hours) as a numerator and the number of nursing professionals effectively working in the 24 hours as a denominator. To identify the number of nursing professionals working in the units, the slack scales (printed format) with the absence notes were verified in a retrospective analysis of the data. Then, the number of nurses and nursing technicians / assistants per day in each unit was determined, composing the number of nursing professionals effectively working on the dates when the APROCENF scale was applied. Thus, the hours required by nursing professionals in the IU and ICU were identified, respectively, according to the equations:

$$\begin{array}{c} {\mathrm{NWLi}}_{\mathrm{IUs}}=\frac{(nº\;\mathrm{MC}\;\times \;4)+(nº\;\mathrm{IC}\;\times \;6)+(nº\;\mathrm{SI}\;\times \;10)+(nº\;\mathrm{HD}\;\times \;10)+(nº\;\mathrm{In}\;\times \;18)}{\mathrm{Quantitative}\;Ν\mathrm{ur}\sin g\;\mathrm{professionals}\;\mathrm{effectively}\;\mathrm{working}\;\mathrm{within}\;24\;\mathrm{hours}}\\ {\mathrm{NWLi}}_{\mathrm{ICUs}}=\frac{\Sigma \mathrm{NAS}}{\mathrm{Quantitative}\;Ν\mathrm{ur}\sin g\;\mathrm{professionals}\;\mathrm{effectively}\;\mathrm{working}\;\mathrm{within}\;24\;\mathrm{hours}} \end{array}$$

The data was organized in Excel® spreadsheet (Win7 Home Basic) and the best care product (“good” and “optimal” score) obtained in the IUs and ICUs was associated with the NWL and NWLi values of these units. 

Statistical analysis was performed using SAS System for Windows (Statistical Analysis System), version 9.2. (SAS Institute Inc, 2002-2008, Cary, NC, USA); The significance level adopted for the tests was 5%, ie p <0.05.

Frequency tables and descriptive statistics with mean, standard deviation, minimum and maximum values, median and quartiles were adopted to describe the sample profile, according to the study variables. For the comparison of categorical variables, the chi-square or Fisher’s exact test (for expected values less than five) were used, and for the numerical variables, the Mann-Whitney test (two categories) and Kruskal-Wallis (three or more categories). In the relationship between numerical variables, the Spearman correlation coefficient was adopted, considering values of low magnitude (0.10 to 0.30), moderate (between 0.4 and 0.6) and strong magnitude (over 0.7)^(^ 20 ^)^. 

In identifying a NWL and NWLi cut-off as predictors of the good and optimal Nursing care product score, the receiver operating characteristic curve (ROC) analysis was used, maximizing sensitivity and specificity and obtaining the area under the curve, which represents the overall performance of the test - the closer to 1.0 (one), the greater the power of the test to discriminate between two groups^(^ 21 ^)^. 

## Results

The evaluators (n = 19) were mostly female (84.2%), with a mean age of 32.4 (SD = 5.4) years and an average length of professional practice of 5.3 (SD = 3.2) years. They performed the role of clinical nurse (68.4%), executive (26.3%) - performing six hours of assistance and the rest in administrative activities - and resident nurse (5.3%). Predominantly, they were specialists (94.7%) in different areas (Cardiology, Degree, Obstetrics, Nephrology, Hospital Management and others), 57.9% were allocated in ICUs - two Medical-Surgical clinics, one Surgical and one Pediatric - and 42.1% in General, Coronary, Pediatric and Neonatal ICUs.

A total of 308 nursing care product evaluations were performed in the ICUs (n = 150) and ICUs (n = 158) in the different shifts - morning (n = 72), afternoon (n = 166) and night (n = 70) higher frequency in the afternoon shift in ICUs (65.2%), prevailing the good score (68.2%). Among the UIs, Pediatrics obtained significant value in the “optimal” classification and the Surgical Clinic, in the “regular” score; In the comparison between ICUs, the Coronary Care Unit presented the highest frequency of “optimal” assessments and the general Intensive Care Unit stood out regarding the “regular” care product (Table 1).

**Table 1:** Classification and comparison between care product scores in hospital units. Campinas, SP, Brazil, 2014 and 2017 (N = 308)

Units	Poor	Regular	Good	Optimal	Total
	N(%)	N(%)	N(%)	N(%)	N(%)
IU* (n=150)					
Medical-Surgical 1	0(0.0)	5(11.9)	36(85.7)†	1(2.4)	42(28.0)
Medical-Surgical 2	1(1.8)	12(22.2)	39(72.2)†	2(3.7)	54(36.0)
Surgical	0(0.0)	13(46.4)†	14(50.0)	1(3.6)	28(18.7)
Pediatrics	0(0.0)	3(11.5)	12(46.1)	11(42.3)†	26(17.3)
Total	1(0.7)	33(22.0)	101(67.3)	15(10.0)	150(100)
ICU‡ (n=158)					
ICU‡ Coronary	1(3.0)	5(15.1)	20(60.6)	7(21.2)†	33(20.9)
ICU‡ Pediatric	0(0.0)	8(15.7)	35(68.6)†	8(15.7)	51(32.3)
ICU‡ General	2(3.7)	11(20.3)†	41(75.9)†	0(0.0)	54(34.2)
ICU‡Neonatal	0(0.0)	1(5.0)	13(65.0)	6(30.0)	20(12.6)
Total	3(1.9)	25(15.8)	109(69.0)	21(13.3)	158(100)

* IU = Inpatient Unit;

† Fischer’s exact test (p≤0.01);

‡ ICU = Intensive Care Unit

The best evaluated items (summing up the grades “3” and “4”) in the IUs and ICUs were: Meeting the care needs (83.4%); Nursing staff sizing (82.5%); Attention to the patient and / or family member (77.6%) and Monitoring and transfer of care (77.3%). Among those with the highest classification number “1” and “2”, the following stand out: Interaction and multidisciplinary action (52.3%); Educational actions and professional development (27.3%); Nursing care planning (23.7%) and Resources needed to provide care (23.7%).

In ICUs, the score of the items Nursing care planning, Nursing staff sizing, Monitoring and transfer of care (p≤0.01) and the care product score (p≤0.05) was higher than in the IUs Nursing staff and NWL were also more representative (p≤0.01) in ICUs.

The NWL of the IUs ranged from 98 (Pediatric) to 240 (Medical-Surgical 2) hours, with averages of 140 (SD = 19.5) hours in Pediatrics, 145 (SD = 15) hours in Medical-Surgical Clinic 1, 157 (SD = 22.4) hours in the Surgical Clinic and 182 (SD = 23.6) hours in the Medical Surgical Unit 2. In the ICUs, the range was 64 (Pediatric ICU) to 528 (General ICU) hours, with mean values of 145 (SD = 42) hours in the Pediatric ICU, 164 (SD = 27.9) hours in the Coronary ICU, 315 (SD = 91.1) hours in the Neonatal ICU and 361 (SD = 71.5) hours in the General ICU. The NWLi ranged from 6.7 (Pediatrics) to 15 (Medical-Surgical 2) and from 3.8 (Pediatric ICU) to 15.5 (Neonatal ICU) hours / professional. In the UIs, the average hours devoted by professional were 9.2 (SD = 1.4) in Pediatrics, 9.6 (SD = 1.8) in Surgical Clinic, 9.7 (SD = 1.1) in Clinic Medical Surgical Unit 1 and 10.3 (SD = 1.9) in the Medical Surgical Unit 2. 

In the IUs, higher values were identified in the number of patients, number of beds, occupancy rate and hours devoted by nursing professionals - NWLi (p≤0.01). These findings are presented in Table 2. 

**Table 2:** Comparison of numerical variables between inpatient units and Intensive Care Units. Campinas, SP, Brazil, 2014 and 2017 (N = 308)

Variables	Inpatient Units (n=150)		Intensive Care Units (n=158)		p‡
	M(SD)*	Md(Q1-Q3)†	M(Dp)*	Md(Q1-Q3)†	
Nursing					
Nurses	3(0.7)	3(3-3)	5.2(2.4)	4(3-7)	≤0.01
Technitians/Auxiliaries	13.4(1.6)	13(12-14)	24.7(11.1)	19(16-36)	≤0.01
Total	16.4(1.9)	16(15-17)	29.9(13.4)	23(19-44)	≤0.01
Patients	22.1(4.2)	22(18-26)	11.5(4.8)	10(8-15)	≤0.01
Beds	27.4(2.5)	29(24-30)	17.2(8.2)	12(10-30)	≤0.01
Occupation(%)	80.7(12.8)	83(73-90)	71.3(19.4)	70(56-90)	≤0.01
Classif patients§					
Minimum	1.8(2.0)	1(0-3)	-	-	
Intermediary	14.6(5.2)	15(11-20)	-	-	
High dependence	1.3(3.0)	0(0-0)	-	-	
Semi-intensive	3.4(2.5)	3(1-5)	-	-	
Intensive	1.0(1.1)	1(0-1)	-	-	
NAS (hours)ǁ	-	-	244(115)	196(155-347)	
NWL (hours)¶	157(27)	156(138-176)	244(115)	196(155-347)	≤0.01
NWLi (hours/prof)**	9.8(1.6)	9.8(8.7-10.7)	8.3(2.3)	7.8(6.8-9.8)	≤0.01
APROCENF††					
Planning	2.9(0.7)	3(3-3)	3.2(0.9)	3(3-4)	≤0.01
Resources	3.0(0.7)	3(3-3)	2.9(0.9)	3(3-3)	
Sizing	2.9(0.8)	3(3-3)	3.2(0.9)	3(3-4)	≤0.01
Education	3.0(0.7)	3(3-4)	2.9(0.9)	3(2-4)	
Monitoring	2.8(0.7)	3(2-3)	3.2(0.8)	3(3-4)	≤0.01
Interaction	2.5(0.8)	2(2-3)	2.4(0.9)	2(2-3)	
Atention	2.9(0.6)	3(3-3)	3.0(0.8)	3(3-4)	
Necessities	3.2(0.6)	3(3-4)	3.1(0.8)	3(3-4)	
Total Score	23.2(3.8)	24(21-26)	24(4.1)	24(22-27)	≤0.05

* M(SD) = Mean and Standard Deviation;

† M (Q1-Q3) = Median and Quartiles;

‡ p = Mann-Whitney test;

§ Classif patients = Classification of patients;

ǁ NAS = Nursing Activities Score;

¶ NWL= Nursing Workload;

** NWLi = Indicator Nursing Workload (hours / professional);

†† APROCENF = Nursing Care Product Evaluation

When comparing the subgroups of the score “good and optimal” versus “regular and poor”, a difference in the UIs over the average patient (p≤0.05), occupation of the units (p≤0.05), NWL (p≤0.01) and NWLi (p≤0.05) was found. No differences were found between these groups in ICUs. 

Spearman’s test (r) showed that there is an inverse relationship between the care product score and the number of patients (-0.19 IUs and -0.24 ICUs), occupancy rate (-0.28 IUs), NWL (-0.25 IUs and -0.18 ICUs) and NWLi (-0.19 IUs and -0.18 ICUs).

In the Medical-Surgical Unit 2 (r = 0.49) and the Neonatal ICU (r = 0.55), the product delivered by Nursing was related, respectively, to the number of technicians and assistants available and the number of nurses (p≤ 0.01). Among APROCENF items, in the IUs, Nursing Care Planning (r = - 0.40) and, in ICUs, Meeting of care needs (r = - 0.41) correlates with the number of hospitalized patients (p≤0.01), as shown in Table 3. 

**Table 3:** Spearman’s correlation coefficient between numerical variables, items and care product score of inpatient units and Intensive Care Units. Campinas, SP, Brazil, 2014 and 2017 (N = 308)

Variables	Nur*	T/A†	Prof‡	Ptes§	Beds	Occupǁ	CTE¶	iCTE**
IU†† (n=150)								
Planning	0.14	-0.17‡‡	-0.20‡‡	-0.40§§	-0.20‡‡	-0.45§§	-0.38§§	-0.21‡‡
Resources	-0.12	-0.32§§	-0.31§§	-0.26‡‡	-0.28‡‡	-0.17‡‡	-0.22‡‡	0.04
Sizing	-0.03	0.19‡‡	0.15	0.01	0.05	-0.07	-0.04	-0.12
Education	-0.09	0.14‡‡	0.08	-0.03	0.09	-0.12	-0.12	-0.17
Monitor	-0.13	-0.03	-0.06	-0.15	0.01	-0.23§§	-0.19‡‡	-0.16
Interaction	-0.10	0.23§§	0.17‡‡	-0.07	0.37§§	-0.30§§	-0.08	- 0.23§§
Atention	-0.13	-0.06	-0.09	-0.24§§	0.00	-0.34§§	-0.28§§	- 0.21§§
Necessities	-0.04	-0.18‡‡	-0.17‡‡	-0.22§§	-0.14	-0.20‡‡	-0.25§§	-0.10
Total Scǁǁ	-0.13	-0.01	-0.05	-0.19‡‡	0.01	-0.28§§	-0.25§§	-0.19‡‡
CMS Sc¶¶1	0.15	-0.31‡‡	-0.20	0.23	-	0.23	0.05	0.15
CMS Sc¶¶2	-0.04	0.49§§	0.38§§	-0.06	-	-0.06	-0.41§§	-0.43§§
SurC Sc***	-0.27	-0.17	-0.24	-0.25	-	-0.25	-0.39‡‡	-0.14
Ped Sc†††	0.09	0.19	0.23	0.24	-	0.24	0.29	0.08
ICU‡‡‡ (n=158)								
Planning	0.19‡‡	0.22§§	0.21§§	0.01	0.19‡‡	-0.34§§	0.14	-0.13
Resources	0.01	0.02	0.03	-0.10	0.03	-0.16‡‡	-0.12	-0.26§§
Sizing	-0.25§§	-0.29§§	-0.28§§	-0.21‡‡	-0.32§§	0.18‡‡	-0.29§§	-0.10
Education	-0.25§§	-0.33§§	-0.32§§	-0.34§§	-0.39§§	0.04	-0.30§§	-0.10
Monitor	-0.27§§	0.33§§	0.32§§	0.15	0.32§§	-0.32§§	0.21§§	-0.12
Interaction	-0.10	-0.11	-0.10	-0.15	-0.08	-0.10	-0.06	-0.03
Atention	-0.12	-0.16‡‡	-0.16‡‡	-0.23§§	-0.17‡‡	0.01	-0.09	0.06
Necessities	-0.32§§	-0.34§§	-0.34§§	-0.41§§	-0.37§§	0.02	-0.27§§	0.04
Total Scǁǁ	-0.13	-0.13	-0.13	-0.24*	-0.15	-0.11	- 0.18‡‡	- 0.18‡‡
COU Sc§§§	-0.24	0.06	0.02	0.17	-	0.02	-0.01	-0.02
PICU Scǁǁǁ	-0.22	-0.14	-0.19	-0.33‡‡	-	-0.32‡‡	-0.34‡‡	-0.31‡‡
GICU Sc¶¶¶	0.08	0.19	0.18	0.10	-	0.09	0.05	-0.04
NICU Sc****	0.55§§	-0.36	-0.09	-0.39	-	-0.39	-0.44	-0.40

* Nur = Nurse;

† T / A = Nursing Technician and Assistant;

‡ Prof = Total Nursing Professionals;

§ Pctes = Patients; CupOcup = Occupancy Rate;

¶ NWL = Nursing Workload;

** NWLi = Indicator Nursing Workload (hours / professional);

†† IU = Inpatient unit;

‡‡ p≤0.05;

§§ P≤0,01;

ǁǁ Total SC = Total Score;

¶¶ CMS Sc = Clinical medical-surgical score;

*** SurC sc = Surgical Clinical Score;

††† Ped Sc = Pediatric Score;

‡‡‡ ICU = Intensive Care Unit;

§§§ COU Sc = Coronary Unit Score; PICU Sc = Pediatric Intensive Care Unit Score;

¶¶¶ GICU Sc = General Intensive Care Unit Score;

**** NICU Sc = Neonatal Intensive Care Unit Score

**Figure 1:**
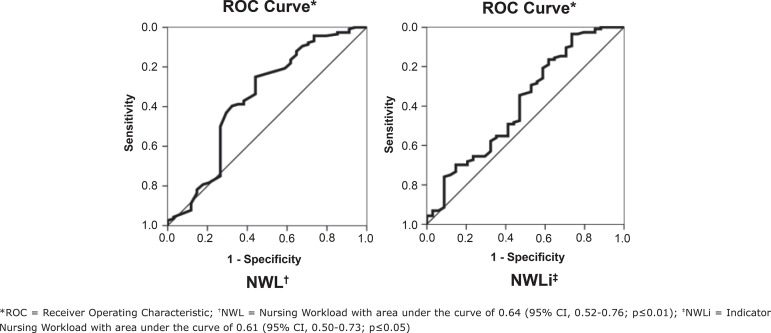
Analysis of ROC^*^ curve in inpatient units for NWL^†^ and NWLi^‡^. Campinas, SP, Brazil, 2014 and 2017 ^*^ROC = Receiver Operating Characteristic; ^†^NWL = Nursing Workload with area under the curve of 0.64 (95% CI, 0.52-0.76; p≤0.01); ^‡^NWLi = Indicator Nursing Workload with area under the curve of 0.61 (95% CI, 0.50-0.73; p≤0.05)

In the ICUs, cut-off points for NWL ≤ 173.0 hours and NWLi ≤ 12.3 hours / nursing professional (Table 4) were associated with a higher probability of obtaining a “good” and “optimal” score in the care product and areas under the curve, respectively, of 0.64 and 0.61 (p ≤0.05) (Figure 1). 

**Table 4:** Results of the ROC* curve for Nursing Workload and indicator Nursing Workload as predictors of the good and optimal care product score, between units and in general. Campinas, SP, Brazil, 2014 and 2017

Predictors	Sensi† (%)	Spec‡ (%)	Cut-off (hours)	PPV§ (%)	NPVǁ (%)	Accuracy (%)
NWL¶						
IUs** (n=150)††	75.0	55.9	≤ 173.0	85.3	39.6	70.7
ICUs‡‡ (n=158)	63.8	50.0	≤ 260.0	85.6	22.9	61.4
Total (N=308)	71.9	38.7	≤ 195.4	82.3	25.8	65.3
NWLi§§						
IUs** (n=150)ǁ	96.6	26.5	≤ 12.3	81.7	69.2	80.7
ICUs‡‡ (n=158)	38.5	82.1	≤ 7.2	90.9	22.3	46.2
Total (N=308)	73.6	40.3	≤ 10.2	83.0	27.8	66.9

* ROC = Receiver Operating Characteristic;

† Sensi = Sensitivity;

‡ Spec = Specificity;

§ PPV = Positive Predictive Value;

ǁ NPV = Negative Predictive Value;

¶ NWL = Nursing Workload;

** IU = Inpatient Units;

†† p≤0.01;

‡‡ ICUs = Intensive Care Units;

§§ NWLi = Indicator Nursing Workload; ≤p≤0.05

## Discussion

This study aimed to present a proposal to associate NWL with a qualitative dimension. The measurement of the workload, as it is known, makes it possible to establish the hours of nursing care through the application of instruments and/or scales available for various practice scenarios and, thus, enabling the team sizing. However, NWL values are numeric and are not associated with outcomes of care delivery. The evaluation of these results makes it possible to improve the care provided and, also, has been used by health care funders as a reward to institutions that offer quality care^(^ 22 ^)^. 

The qualitative look at the workload has not been properly explored in the literature. A study conducted in Finland more than a decade ago is highlighted, that proposed a method to estimate the best level of nursing care intensity, contributing to the allocation of resources to meet the needs of patients^(^ 23 ^)^. More recently, this assessment has been made up of a system implemented in almost every hospital in this country along with the daily patient classification, the number of available nursing staff and financial information^(^ 24 ^)^. 

Thus, the construction of an NWLi for alignment with the APROCENF scale, which had its psychometric properties tested in Brazil, was sought^(^ 15 ^)^. Through the interaction between structural factors and work organization methods that intervene in the care process, this scale instrumentalizes the nurse manager in the identification of critical points in the units^(^ 15 ^)^. 

In order to reach the proposed objectives, APROCENF was applied in different hospital units (IUs and ICUs) and, in the nurses’ evaluation of shifts (N = 308), the delivered product was mostly good (68.2%), with better ICU score. Validation study of this scale^(^ 15 ^)^ and another performed in specialized hospitals^(^ 25 ^)^ also identified good care product - 64.5% and 69.5%, respectively. The findings make it possible to infer that in highly specialized services^(^ 25 ^)^ and those where patients require high clinical dependence, material conditions, available resources and work organization may favor the care delivered by nursing. 

It was also possible to recognize that the qualifying factors of this product in the IUs and ICUs, that is, the best scored items were: Meeting the care needs and Nursing staff sizing, also pointed out in previous investigations^(^ 15 ^,^ 25 ^)^. On the other hand, the inter-professional action was critical in the production of care in these units and corroborates other studies^(^ 15 ^,^ 25 ^)^. This lack of collaboration among health professionals has been the object of worldwide research and debate proposing interventions in training and in the workplace to improve practice and care^(^ 26 ^-^ 27 ^)^. 

There was also an inverse correlation between the care product score, NWL and NWLi, although weak, in the IUs and ICUs, and in the Medical-Surgical Unit 2 and Neonatal ICU, this association was moderate. It is noteworthy that the Medical-Surgical clinic 2 had a high workload (average of 182, reaching up to 240 hours) and each nursing professional would need to devote more time (up to 15 hours) to patient care on some evaluated days, as well as in the neonatal ICU (up to 15.5 hours / professional). This Medical-Surgical Unit also showed a positive correlation between the delivered product and the number of technicians and assistants, as well as, in the Neonatal ICU, the correlation between the score and the number of nurses was positive and moderate. 

Work overload has been associated with unwanted care delivery events (falls, medication errors and infections) as well as situations that predispose to occupational dropout (exhaustion and job dissatisfaction)^(^ 9 ^)^. But so far, no studies have been identified that correlate the NWL, the hours devoted by professional and the product of care, and further research is needed to test the data found.

In IUs, specifically, a significant relationship was found between the product score of “regular and poor” care and higher average patients and occupancy rate, high NWL and dedicated / professional hours. Also, in this study, the number of patients, unit occupancy and NWLi were significantly higher than in ICUs. These IUs, at the rear of an overcrowded emergency service, have a high demand for care, maintaining occupancy of over 80% and patients requiring semi-intensive and intensive care. Another study also identified the same profile of patients in ICUs^(^ 28 ^)^. 

The high number of patients attributed to the IU Nursing team negatively impacts the safety of patients and professionals^(^ 29 ^)^. There is also a optimaler loss of productivity due, among others, to the physical and functional structure and the difficulty of monitoring the activities performed^(^ 30 ^)^. 

In this study, it was demonstrated that the number of patients inversely interferes with the planning of care delivered by the nurses of the IU and the care of care needs in ICUs. In the IUs, the formalized care plan based on clinical assessment and comprehensive care may be neglected to the detriment of the number of patients to be assisted. This process may be compromised by prescribing routine / standard actions that do not meet individual needs^(^ 31 ^)^ or lack of records and, therefore, lack of legal support to professionals^(^ 32 ^)^. 

In ICUs, the more inpatients, the less projected interventions are performed. This fact is a warning for nursing practice, as planned care is not fully implemented, considering increasingly complex units and operating at their maximum capacity. 

A study conducted in ICUs of Iceland^(^ 33 ^)^ also showed a positive but weak correlation between the number of patients and omission of care, that is, the more patients, the more activities may be missed. In addition, it also found that adequacy in staff sizing and improved teamwork diminish occurrences of missed or delayed care. Australian researchers^(^ 27 ^)^ ratify this relationship between strengthened teamwork and fewer forgotten care with better outcomes in ICUs, probably due to the proportion of patients per nurse. 

A cut-off point was also obtained of NWL ≤ 173 hours and NWLi ≤ 12.3 hours / Nursing professional as predictors of the “good” and “optimal” care product score in the IU. Of the four IUs investigated, only Medical-Surgical Clinic 2 had an average NWL higher than the cut-off value - 182 hours. Regarding the NWLi, on average, no unit reached the cut-off point, however, the Medical-Surgical Clinic 2 presented values of up to 15 hours / professional and, of the 54 evaluations performed in this unit, 11 (20.4%) exceeded 12.3 hours / Nursing professional. This aspect deserves attention, since the exposure of the team to values above the cut, in some days, incurs risks or deficiencies in the delivery of care. In other words, high burdens have a negative impact on the qualitative dimension of the practice environment (available resources, in-service education, teamwork), care planning and follow-up, patient / family care, meeting needs and prevention of incidents. 

Because it is the first national investigation associating NWL and NWLi with a product evaluation delivered by Nursing, the study has limitations. The NWL and NWLi cut-offs found are preliminary data and were constructed from a practice scenario. It has not yet been possible to determine at this time what the cut-off for ICUs would be, and cut-off points for the regular and poor care product have not been tested. Therefore, this proposal needs to be implemented in new scenarios to verify if these values are confirmed. 

The association of NWLi and APROCENF enables managers to identify how NWL is interfering with the care product, enabling each service to establish its own standards, making the necessary adjustments according to a given reality. In addition, it favors to investigate the production of care more broadly, identifying points of improvement. Thus, nurses would have subsidies to manage care, considering the needs of patients / families and the nursing team from the perspective of workload and delivery of the best care product. Therefore, this proposal instructs nurses in the allocation of human capital compatible with the demand for attention and in the adjustment of resources and processes crucial for nursing to develop qualified work.

## Conclusion

The proposal to associate workload and its indicator with a qualitative dimension is feasible. NWL cut-off point ≤ 173 hours and NWLi ≤ 12.3 hours / professional were predictors of the “good” and “optimal” nursing care product score in inpatient units. 

These findings aim to contribute to the hospital management and nursing care systems, seeking to balance work demands, working conditions, quality of care and cost-effectiveness of the service.
